# Plantar flexor strength and size decrease following single‐leg disuse in uninjured adults: A meta‐analysis

**DOI:** 10.1111/cpf.12912

**Published:** 2024-11-04

**Authors:** Nicholas Preobrazenski, Joel Seigel, Ian Janssen, Sandra Halliday, Chris McGlory

**Affiliations:** ^1^ Faculty of Medicine University of Ottawa Ottawa Ontario Canada; ^2^ School of Kinesiology and Health Studies Queen's University Kingston Ontario Canada; ^3^ Department of Public Health Sciences Queen's University Kingston Ontario Canada; ^4^ Queen's University Library Queen's University Kingston Ontario Canada; ^5^ Department of Medicine Queen's University Kingston Ontario Canada

**Keywords:** calf muscle, computed tomography, human, immobilisation, magnetic resonance imaging, muscle disuse, unloading, unweighting

## Abstract

**Introduction:**

Plantar flexors play a pivotal role in human locomotion and balance. Several original research studies and systematic reviews have characterised the impact of single‐leg disuse on plantar flexor strength and size. However, no meta‐analysis has quantified the effects of single‐leg disuse on changes in plantar flexor strength and size in uninjured adults.

**Aim:**

To quantify changes in plantar flexor strength and size in response to single‐leg disuse.

**Methods:**

Data were extracted from 19 studies captured in our previous systematic review on studies that employed a unilateral lower limb immobilisation model (cast or brace) and were published up to January 30, 2022. Random‐effects meta‐analyses were performed on original research studies reporting measures of plantar flexor strength (isometric, isokinetic, or repetition maximum) and size (magnetic resonance imaging or computed tomography) in uninjured adults.

**Results:**

Single‐leg disuse decreased plantar flexor strength (Hedges *g*_av_ = −0.71 [95% confidence interval: −0.93, −0.48], *p* < 0.001, 7−28 days, *N* = 16 studies, *n* = 121 participants including ≥13 females, ages 19−29) and plantar flexor size (−0.33 [−0.50, −0.15], *p* < 0.001, 14−35 days, *N* = 6, *n* = 49, 10 females, ages 22−27) across all durations of disuse.

**Discussion:**

Single‐leg disuse decreases plantar flexor strength and size in uninjured adults. This work adds to recent meta‐analytic findings demonstrating the declines in knee extensors strength and size following single‐leg disuse. The paucity of female and participants >30 years old in the single‐leg disuse literature examining plantar flexors represents a priority of future work.

## 1 INTRODUCTION

The plantar flexors are a group of skeletal muscles in the lower limb that enable functional movements such as walking, running, and jumping. The plantar flexors also influence gait speed, (Tavakkoli Oskouei et al., 2021) enhance venous return via the venous pump, (Ludbrook, 1966) and play a pivotal role in maintaining balance (Onambele et al., 2006). Importantly, plantar flexor strength is an essential contributor to maintaining balance and mitigating the risk of falls in older adults (Namayeshi et al., 2023; Pijnappels et al., 2005).

We (Mcglory et al., 2019; Preobrazenski, Seigel, et al., 2023) and others (Campbell et al., 2019; Hardy et al., 2022; Wall & van Loon, 2013) have shown that single‐leg disuse leads to a rapid decline in skeletal muscle strength and size. Loss of skeletal muscle size and strength increases the risk of disability, metabolic disease, and falls (Bell et al., 2016; Horlings et al., 2008; Janssen & Ross, 2005)—the latter being the second leading cause of accidental deaths worldwide (Roth et al., 2018). Although most single‐leg disuse studies have focused on the knee extensors, (Adams et al., 2003; Campbell et al., 2019; Hardy et al., 2022; Preobrazenski, Seigel, et al., 2023; Wall and van Loon, 2013) research has also shown that the plantar flexors undergo significant declines in strength (Fitts et al., 2000) and size (Fitts et al., 2000; Hardy et al., 2022) in response to single‐leg disuse. Bed rest studies further support these findings by showing that declines in muscle strength and size can occur more rapidly in the plantar flexors compared with the knee extensors (Alkner & Tesch, 2004; Hardy et al., 2022).

To our knowledge, only two systematic reviews have examined changes in plantar flexor strength (Campbell et al., 2019) and/or size (Campbell et al., 2019; Hardy et al., 2022) following single‐leg disuse in uninjured adults, and no meta‐analysis has assessed the impact of single‐leg disuse on plantar flexor strength. Meta‐analyses increase statistical power and provide more precise estimates of effect size by synthesising data from multiple studies. A meta‐analysis that quantifies changes in plantar flexor strength and size in response to single‐leg disuse would provide novel data for the literature. Therefore, the aim of the present study was to meta‐analyse the effects of single‐leg disuse on plantar flexor strength and size in adult women and men.

## 2 METHODS

Eligibility criteria, search strategies, selection process, data extraction, and data synthesis methods used to generate the list of studies analysed in the current study have been described previously (Preobrazenski, Seigel, et al., 2023). Eligible studies were original experimental research involving non‐injured human participants, employing a unilateral leg immobilisation model, and reporting skeletal muscle size, strength, or power data following a period of single‐leg disuse without countermeasures (e.g., concomitant exercise training). Studies were excluded if they did not meet all inclusion criteria, were not published in English, reported previously published skeletal muscle data, or if the full texts were unavailable despite multiple library searches and attempts to contact the authors. The current work provides additional analyses of previously extracted, unanalysed data. Studies were included in the current meta‐analyses if they reported plantar flexor strength and size data from uninjured adults who underwent single‐leg disuse (osf.io/wdexu; osf.io/ut93s).

To extract plantar flexor strength and size data, two authors (N. P. and J. S.) independently revisited all studies included in our previous systematic review (Preobrazenski, Seigel, et al., 2023). The current meta‐analysis included studies that reported or provided extractable baseline and postintervention mean and standard deviation/standard error data for plantar flexor strength and size of the immobilised and non‐immobilised legs. WebPlotDigitizer (https://automeris.io/WebPlotDigitizer/) was used when outcome data only appeared in figures. Random‐effects model meta‐analyses, forest plots, and funnel plots were conducted using Meta‐Essentials software version 1.4. Forest plots present Hedges' *g*_*av*_ with 95% confidence intervals (CI) and prediction intervals. ﻿Effect sizes were interpreted as trivial (g_*av*_ < 0.2), small (*g*_av_ ≥ 0.2), medium (*g*_av_ ≥ 0.5), or large (*g*_av_ ≥ 0.8) (Cohen, 1992). Heterogeneity and bias assessment methods were identical to our previous meta‐analyses on leg extensor strength and size declines following single‐leg disuse (Preobrazenski, Seigel, et al., 2023; Preobrazenski, Janssen, et al., 2023).

## 3 RESULTS

**Figure 1:**
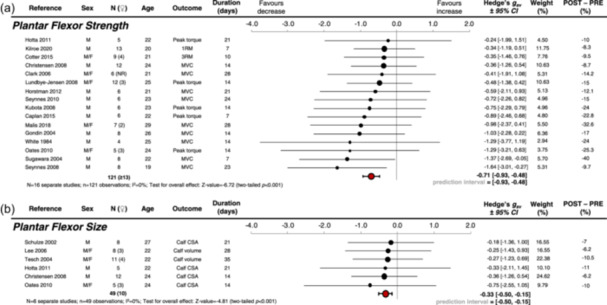
Forest plot of a random effects meta‐analysis of within‐subject changes in (a) plantar flexor strength and (b) size of the immobilised leg of noninjured adults in single‐leg disuse studies. Because effect sizes were calculated as baseline minus postdisuse values, negative Hedge's *g* values with larger magnitudes reflect larger declines in plantar flexor strength or size. Red circles represent combined effect sizes with 95% confidence intervals. Outcomes reported in N, kg, and Nm were categorised as MVC, 1RM, and peak torque, respectively. *N* = 19 unique studies. CI, confidence interval; CSA, cross‐sectional area; MVC, maximum voluntary contraction; NR, not reported; 1RM, one‐repetition maximum; 3RM, three‐repetition maximum.

Of the 86 studies from our systematic review on single‐leg disuse, (Preobrazenski, Seigel, et al., 2023) data from the immobilised leg of 19 studies were included in the current meta‐analyses (Figures 1a,b) (Caplan et al., 2015; Christensen et al., 2008; Clark et al., 2006; Cotter et al., 2015; Gondin et al., 2004; Horstman et al., 2012; Hotta et al., 2011; Kilroe et al., 2020; Kubota et al., 2008; Lee et al., 2006; Lundbye‐Jensen & Nielsen, 2008; Malis et al., 2018; Oates et al., 2010; Schulze et al., 2002; Seynnes et al., 2008; Seynnes et al., 2010; Sugawara et al., 2004; Tesch et al., 2004; White et al., 1984). Supporting Information S1: Sheet S2 presents included study details, and Supporting Information S1: S3 presents all extracted data. Supporting Information S1: Sheet S5 presents excluded studies and reasons for exclusion. Sixteen studies provided plantar flexor strength data, (Caplan et al., 2015; Christensen et al., 2008; Clark et al., 2006; Cotter et al., 2015; Gondin et al., 2004; Horstman et al., 2012; Hotta et al., 2011; Kilroe et al., 2020; Kubota et al., 2008; Lundbye‐Jensen and Nielsen, 2008; Malis et al., 2018; Oates et al., 2010; Seynnes et al., 2008; Seynnes et al., 2010; Sugawara et al., 2004; White et al., 1984) and six studies provided plantar flexor size data (Christensen et al., 2008; Hotta et al., 2011; Lee et al., 2006; Oates et al., 2010; Schulze et al., 2002; Tesch et al., 2004). Of the 19 separate studies (*n* = 148), seven studies (37%) included females (*n* ≥ 20, ≥14%), and all studies reported average participant ages between 19 and 29 years of age.

Twelve studies (63%) reported compliance measures related to single‐leg disuse, (Clark et al., 2006; Cotter et al., 2015; Horstman et al., 2012; Hotta et al., 2011; Kilroe et al., 2020; Lee et al., 2006; Malis et al., 2018; Oates et al., 2010; Schulze et al., 2002; Seynnes et al., 2010; Shin et al., 2008; Tesch et al., 2004) nine studies (47%) reported taking measures to mitigate risk of deep vein thromboses, (Clark et al., 2006; Horstman et al., 2012; Hotta et al., 2011; Kilroe et al., 2020; Lee et al., 2006; Oates et al., 2010; Schulze et al., 2002; Seynnes et al., 2010; Shin et al., 2008; Tesch et al., 2004) and only one study (5%) reported a thrombotic event (Tesch et al., 2004). Three studies (16%) reported performing a priori sample size calculations (Clark et al., 2006; Gondin et al., 2004; Oates et al., 2010).

The funnel plot for plantar flexor strength (Supporting Information S1: Sheet S6) suggested publication bias (Egger's *p* = 0.015) with an adjusted combined effect size of −0.48 [−0.72, −0.24]. Twelve of 19 studies (63%) reported implementing compliance measures related to single‐leg disuse (Supporting Information S1: Sheet S3), and all studies were judged to have overall unclear or high risk of bias (Supporting Information S1: Sheet S4).

Figure 1a presents the forest plot for changes in plantar flexor strength (overall Hedges' g_av_ with 95% CI = −0.71 [−0.93, −0.48], *p* < 0.001, 7−28 days, *N* = 16 studies, *n* = 121 participants). Figure 1b shows the forest plot for changes in plantar flexor size (−0.33 [−0.50, −0.15], *p* < 0.001, 14−35 days, *N* = 6, *n* = 49). The Shapiro−Wilk test indicated a deviation from normal distribution for strength data (*p* = 0.047), but not for size data (*p* = 0.089). The mean (±standard deviation) and median declines in plantar flexor strength across all durations of disuse were −18.0 ± 9.3% and −15.0% (range: −40% to −8.3% (Caplan et al., 2015; Christensen et al., 2008; Clark et al., 2006; Cotter et al., 2015; Gondin et al., 2004; Horstman et al., 2012; Hotta et al., 2011; Kilroe et al., 2020; Kubota et al., 2008; Lundbye‐Jensen and Nielsen, 2008; Malis et al., 2018; Oates et al., 2010; Seynnes et al., 2008; Seynnes et al., 2010; Sugawara et al., 2004; White et al., 1984), respectively. The mean and median declines in plantar flexor size were −8.5 ± 2.3% and −8.5% (−11% to −6.2% [Christensen et al., 2008; Hotta et al., 2011; Lee et al., 2006; Oates et al., 2010; Schulze et al., 2002; Tesch et al., 2004]). Moderator analysis revealed that age was not a predictor of declines in plantar flexor strength (*β* = −0.13, *p* = 0.70) or size (*β* = 0.34, *p* = 0.80).

Meta‐analyses of a small subset of studies that reported data from both the immobilised and nonimmobilized lower leg revealed no changes in plantar flexor strength (0.04 [−0.19, 0.27], *p* = 0.56, 7−21 days, *N* = 4, *n* = 34) (Christensen et al., 2008; Hotta et al., 2011; Kilroe et al., 2020; White et al., 1984) or size (0.06 [−0.26, 0.38], *p* = 0.40, 14−35 days, *N* = 3, *n* = 28) (Christensen et al., 2008; Hotta et al., 2011; Tesch et al., 2004) of the non‐immobilised leg following single‐leg disuse. Shapiro−Wilk tests indicated a normal distribution for strength (*p* = 0.30) and a non‐normal distribution for size (*p* = 0.02) of the nonimmobilized leg. The mean and median changes in plantar flexor were 2.7 ± 3.7% and 3.5% (range: −1.9% to 5.7%), respectively. The mean and median percent changes in plantar flexor size were 3.2 ± 4.4% and 0.7% (0.6%−8.3%).

In contrast, there were changes in plantar flexor strength (−0.43 [−0.96, 0.10], *p* = 0.01) (Christensen et al., 2008; Hotta et al., 2011; Kilroe et al., 2020; White et al., 1984) and size (−0.32 [−0.44, −0.19], *p* < 0.001) (Christensen et al., 2008; Hotta et al., 2011; Tesch et al., 2004) of the immobilised leg following single‐leg disuse in studies reporting data from both the immobilised and non‐immobilised lower legs. Shapiro−Wilk tests indicated a non‐normal distribution for strength (*p* = 0.02) and a normal distribution for size (*p* = 0.18) of the immobilised leg. The mean and median changes in plantar flexor strength were −12.8 ± 7.5% and −9.4% (range: −24% to −8.3%). The mean and median percent changes in plantar flexor size were −9.2 ± 2.6% and −10.5% (−11.0% to −6.2%).

## 4 DISCUSSION

The primary findings of the current study are that single‐leg disuse leads to a medium effect size decline in plantar flexor strength and a small effect size decline in plantar flexor size. Furthermore, single‐leg disuse does not result in a decline in plantar flexor strength or size in the nonimmobilised, contralateral leg. This finding extends our previous work from the leg extensors (Preobrazenski, Janssen, et al., 2023) and provides further evidence that the contralateral leg can be used as a valid, within‐subject control to examine changes in plantar flexor strength and size following single‐leg disuse in healthy uninjured adults. However, with only a few studies providing data from both legs (strength: *n* = 4; size: *n* = 3), the statistical power of this analysis is limited, and our findings should be interpreted cautiously.

The changes in plantar flexor strength (−0.71) and size (−0.33) following single leg disuse are similar in magnitude to our previously reported declines in knee extensor strength (−0.80) and size (−0.41) (Preobrazenski, Seigel, et al., 2023). Plantar flexor strength appeared to decrease more than plantar flexor size (−0.71 vs. −0.33), perhaps because disuse leads to decreased motor unit recruitment that impairs force generation even when muscle size remains relatively unchanged (Inns et al., 2022). Our findings also align with previous reviews reporting larger declines in lower limb muscle strength compared with size following disuse in uninjured adults (Adams et al., 2003; Campbell et al., 2019; Marusic et al., 2021; Wall & van Loon, 2013; Wall et al., 2013).

Physical activity levels were not factored into the analyses because only one study objectively tracked and reported physical activity (Clark et al., 2006). Moreover, we included a range of strength measures, including maximal voluntary contraction, one and three repetition maximums, and peak torque which could have introduced additional variability. However, despite the different techniques used to assess muscle strength, our meta‐analyses revealed no heterogeneity among the included studies (*I* ^2^ = 0%), and this supports the overall observation that single‐leg disuse decreases plantar flexor strength. Although the imputed data points in the funnel plot for plantar flexor strength suggested missing reports of increases in strength following single‐leg disuse, disuse should not result in strength gains. Confirming this expectation requires consistently reported compliance measures to ensure adherence to disuse protocols.

Study populations were also relatively homogenous (i.e., ~85% young males). While examining only uninjured young adults mitigates confounding factors that can affect muscle strength and size, such as hypercortisolemia (Fitts et al., 2007) and hyperinflammation, (Jurdana et al., 2015) this approach restricts the generalisability of our findings. Specifically, our findings may not extend to clinical populations who experience larger declines in plantar flexor size following disuse (Hardy et al., 2022) or to groups more adversely affected by skeletal muscle disuse such as females and older adults (Bell et al., 2016; Nunes et al., 2022). Addressing this limitation is challenging because it is ethically complex to subject older adults to immobilisation protocols due to increased risks associated with disuse in this population (Nunes et al., 2022). Consequently, studies involving older adults are scarce, and it was unsurprising that the moderator analysis revealed that age was not a predictor of plantar flexor strength of size given the small age range of studies included in these meta‐analyses. Future single‐leg disuse studies should include understudied populations.

In conclusion, the present meta‐analyses demonstrate that single‐leg disuse reduces plantar flexor strength and size in uninjured adults. Future research should prioritise including women and older adults and ensure the rigorous reporting of compliance and objective physical activity measures. Addressing these priorities can advance our understanding of the effects of disuse on lower limb skeletal muscle to inform the development of effective strategies for prevention and rehabilitation of muscle atrophy and strength loss.

## AUTHOR CONTRIBUTIONS

**Nicholas Preobrazenski**: Led data curation, formal analysis, investigation, visualisation, and original draft writing; contributed equally to conceptualisation, methodology, project administration, software, supervision, validation; and led review and editing. **Joel Seigel**: Contributed equally to data curation and validation; supported formal analysis. **Ian Janssen**: Supported formal analysis and validation; contributed equally to methodology, software, and review and editing. **Sandra Halliday**: Supported data curation, methodology; contributed equally to software and review and editing. **Chris McGlory**: Contributed equally to conceptualisation, project administration, software, supervision, and validation; supported formal analysis, investigation, methodology, visualisation, original draft writing, and review and editing.

## CONFLICT OF INTEREST STATEMENT

The authors declare no conflict of interest.

## Supporting information

Supporting information.

## Data Availability

All data can be found in the Supporting Information accompanying this article.
